# High expression of PTPRM predicts poor prognosis and promotes tumor growth and lymph node metastasis in cervical cancer

**DOI:** 10.1038/s41419-020-02826-x

**Published:** 2020-08-11

**Authors:** Pan Liu, Chunyu Zhang, Yuandong Liao, Junxiu Liu, Jiaming Huang, Meng Xia, Ming Chen, Hao Tan, Weipeng He, Manman Xu, Tianyu Liu, Shiyin Ooi, Qiqiao Du, Shuhang Qin, Yuan Zhu, Qiaojian Zou, Wei Wang, Shuzhong Yao

**Affiliations:** Department of Obstetrics and Gynecology, The First Affiliated Hospital, Sun Yat-sen University, Guangzhou, People’s Republic of China

**Keywords:** Cervical cancer, Metastasis

## Abstract

The prognosis for cervical cancer (CCa) patients with lymph node metastasis (LNM) is dismal. Elucidation of the molecular mechanisms underlying LNM may provide clinical therapeutic strategies for CCa patients with LNM. However, the precise mechanism of LNM in CCa remains unclear. Herein, we demonstrated that protein tyrosine phosphatase receptor type M (PTPRM), identified from TCGA dataset, was markedly upregulated in CCa with LNM and correlated with LNM. Moreover, PTPRM was an independent prognostic factor of CCa patients in multivariate Cox′s proportional hazards model analysis and associated with poor prognosis. Furthermore, through gain-of-function and loss-of-function approaches, we found that PTPRM promoted CCa cells proliferation, migration, invasion, lymphangiogenesis, and LNM. Mechanistically, PTPRM promoted epithelial–mesenchymal transition (EMT) via Src-AKT signaling pathway and induced lymphangiogenesis in a VEGF-C dependent manner, resulting in LNM of CCa. Importantly, knockdown of PTPRM dramatically reduced LNM in vivo, suggesting that PTPRM plays an important role in the LNM of CCa. Taken together, our findings uncover a novel molecular mechanism in the LNM of CCa and identify PTPRM as a novel prognostic factor and potential therapeutic target for LNM in CCa.

## Introduction

Cervicer (CCa) is the fourth most common malignant cancer among females worldwide with approximately 569,847 new cases and 311,365 deaths in 2018^1^. Nearly 90% of cervical cancer occurred in developing countries in 2015^1,2^. Lymph node metastasis (LNM) is a key prognostic factor and the leading cause of death of CCa^3–5^. The 5-year overall survival rate of CCa patients decreases from 95% to 51% if with LNM, even when the CCa is treated with radical hysterectomy^6,7^. However, the mechanisms underlying LNM of CCa have not been fully elucidated.

Protein tyrosine phosphatases (PTPs) are important antagonists of tyrosine kinase dependent signaling pathway and PTPs regulate various aspects of biological processes such as cell proliferation, migration and transformation^8–10^. Protein tyrosine phosphatase receptor type M (PTPRM) is a member of the PTP family and was reported as a tumor-associated factor which was mutated in many kinds of cancers. It has been reported that increased expression of PTPRM was negatively correlated with the progression of colorectal adenoma-carcinoma, small intestinal neuroendocrine tumors and breast cancer^11–13^, while the single-nucleotide polymorphisms of the PTPRM suggested it could play an oncogenic role in lung cancer^14^. However, the role and precise mechanism of PTPRM in CCa remains unknown, warranting further exploration.

In this study, we identified PTPRM which was markedly overexpressed in locally advanced cervical cancer from TCGA dataset. Then we validated the expression of PTPRM in our tissue cohort and found that PTPRM was significantly up-regulated in CCa patients with LNM. Moreover, PTPRM was correlated with LNM and poor prognosis in CCa. Furthermore, overexpression of PTPRM promoted cervical cancer cells proliferation, migration, invasion, epithelial-to-mesenchymal transition (EMT), lymphangiogenesis, and LNM in vitro and in vivo. Mechanistically, PTPRM promoted LNM of CCa through VEGF-C induced lymphangiogenesis and Src-AKT signaling pathway mediated EMT. Our findings reveal a novel molecular mechanism of LNM and identify PTPRM as a prognostic factor and potential therapeutic target for LNM in CCa.

## Results

### Identification of PTPRM in CCa from TCGA dataset

**Fig. 1:**
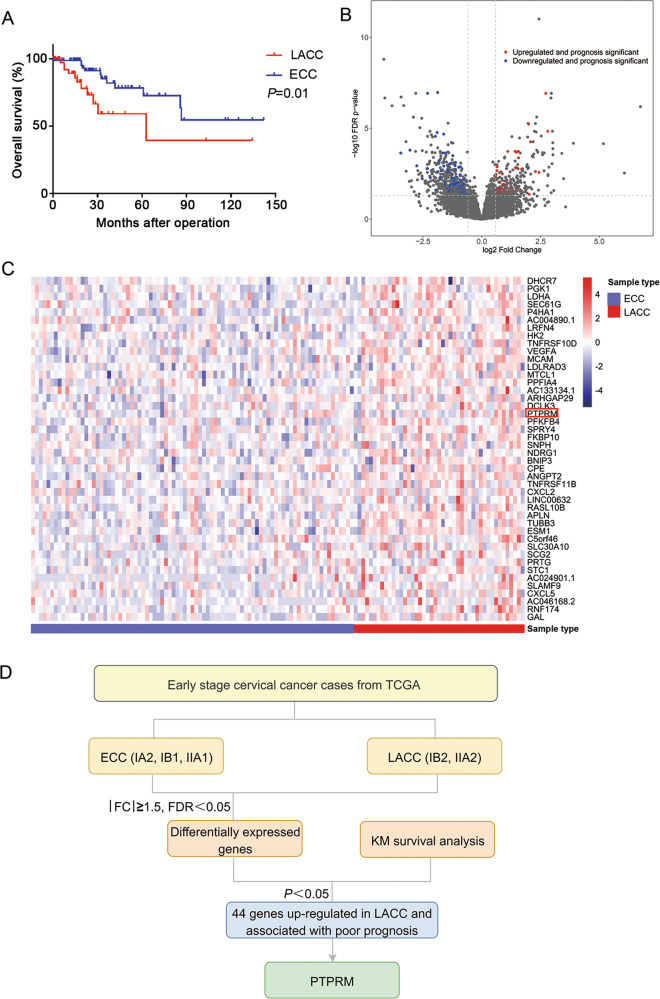
Identification of PTPRM in CCa from TCGA dataset. **a** LACC group patients had a worse OS compared with ECC group in TCGA database. **b** The volcano plot showed the differential expressed genes between LACC and ECC group in TCGA database. **c** Heat map presented 44 genes highly expressed in LACC group and related to the poor prognosis of CCa. **d** The detailed screening flowchart for PTPRM.

We divided the CCa cases (FIGO stage IA2 to IIA) into 2 groups depending on the tumor size: locally advanced CCa (LACC) group (FIGO stage IB2 and IIA2) and early stage CCa (ECC) group (FIGO stage IA2, IB1 and IIA1). Then we analyzed TCGA dataset and found that the overall survival of LACC group patients was significantly worse than the ECC group patients (Fig. 1a). Meanwhile, the volcano plot was performed to show the differentially expressed genes between LACC group tissues and the ECC group tissues (Fig. 1b). We identified 920 differentially expressed genes between ECC group and LACC group. Among these genes, 314 genes were upregulated in LACC tissues, of which 44 genes were correlated with poor survival of CCa and another 646 genes were down-regulated in LACC tissues, of which 132 genes were correlated with poor survival of CCa. GO and KEGG enrichment results of 176 dysregulated genes with prognostic significance were shown in Supplementary Fig. S1a. We identified PTPRM that was upregulated in LACC group tissues and correlated with poor survival of CCa (Fig. 1c). The detailed screening method was shown in Fig. 1d.

### PTPRM is upregulated in CCa with LNM and correlates with LNM and poor prognosis

**Fig. 2:**
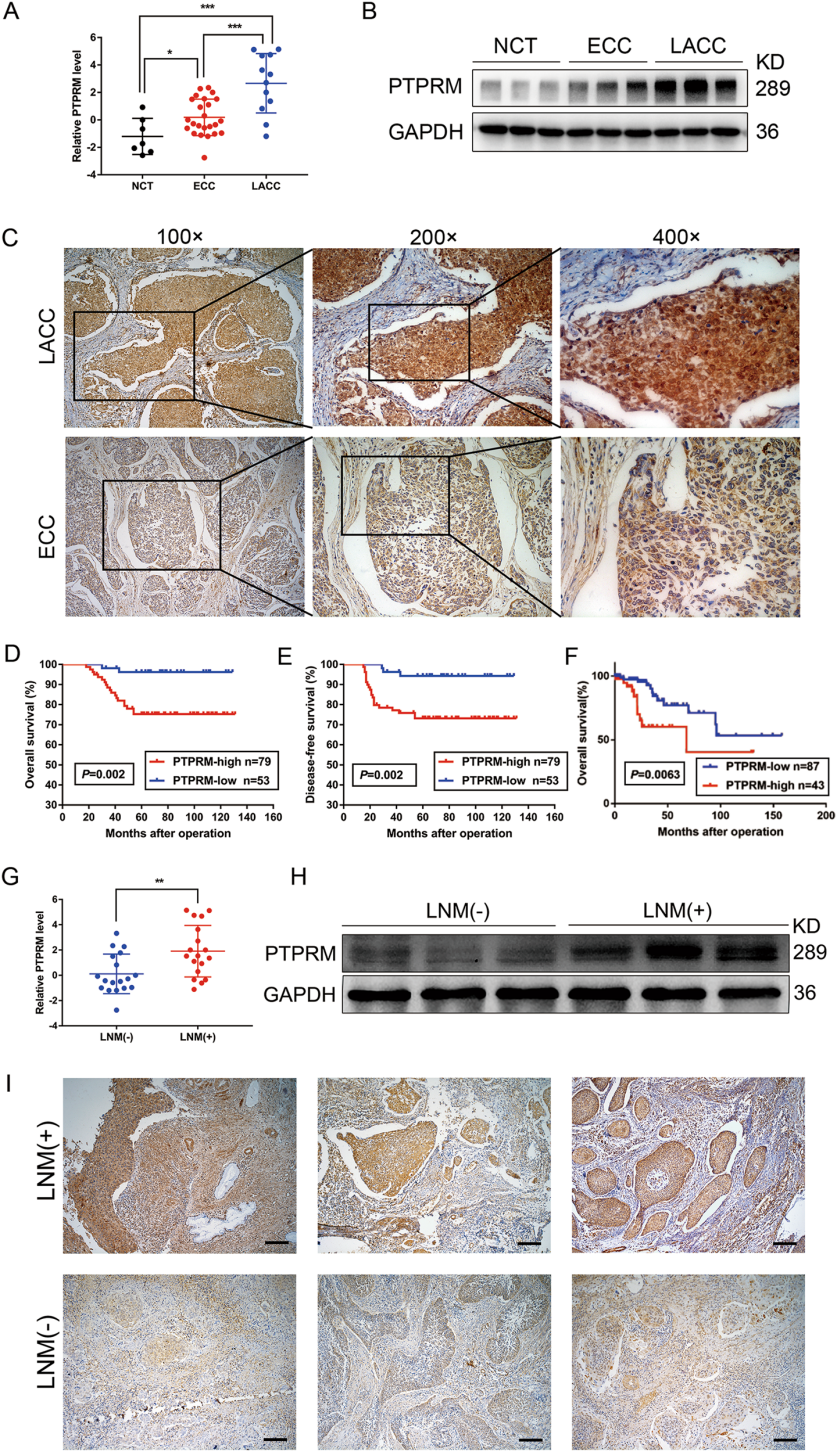
PTPRM is upregulated in CCa with LNM and correlates with LNM and poor prognosis. **a**, **b** The results of qRT-PCR and Western blot showed PTPRM was up-regulated in LACC group patients compared with ECC group and normal cervix tissues (NCT). **c** Representative IHC images confirmed the expression level of PTPRM in ECC and LACC tissues. Original magnification: ×100, ×200, ×400. **d**, **e** CCa patients with high PTPRM level had a poor OS and DFS analyzed in our specimen cohort (IA2-IIA2). **f** TCGA database showed the expression of PTPRM was negatively associated with OS of CCa patients (IA2-IIA2). **g**, **h** qRT-PCR and Western blot demonstrated that PTPRM presented a higher expression in lymphatic metastatic CCa tissues. **i** The representing IHC images of PTPRM in CCa tissues with or without lymphatic metastasis. Original magnification: ×100. *, ** or ***: significantly different from the corresponding control, *P* < 0.05, *P* < 0.01 or *P* < 0.001, respectively.

To validate whether PTPRM is upregulated in LACC, we first tested the expression of PTPRM in our tissues cohort. Our results showed that PTPRM in the LACC tissues was significantly higher than the ECC and normal cervix tissues (NCTs) in both mRNA and protein levels (Fig. 2a, b). To evaluate the association between PTPRM protein expression and clinicopathological factors in CCa, the expression of PTPRM was examined by IHC analysis in 132 CCa tissues. Our data showed that PTPRM was markedly overexpressed in LACC cases compared with the ECC cases (Fig. 2c). Moreover, high PTPRM expression had a notable correlation with tumor size (*P* = 0.019) and LNM (*P* = 0.015) (Table 1). In the multivariate Cox′s proportional hazards model analysis, PTPRM expression level, tumor size, LVSI, and LNM were found to be independent prognostic factors (Table 2). Furthermore, Kaplan-Meier survival analysis and the log-rank test survival analysis showed that the patients with high PTPRM expression had significantly decreased OS and DFS in our CCa tissues cohort and TCGA dataset (Fig. 2d–f). Since PTPRM was significantly associated with LNM in CCa, we tried to investigate the expression of PTPRM in CCa tissues with LNM and without LNM. We demonstrated that PTPRM presented a higher expression in CCa with LNM both in protein and mRNA levels, compared with those without LNM (Fig. 2g–i). Collectively, these results indicate that PTPRM is upregulated in CCa with LNM and correlates with LNM and poor prognosis.

**Table 1:** Correlation between PTPRM expression and clinicopathologic characteristics of CCa.

Characteristics	Total	PTPRM expression		*P-*value
	132	High	Low	
Age				0.166
<44	65	35	30	
≥44	67	44	23	
FIGO stage				0.233
Ia2	6	3	3	
Ib1	94	53	41	
Ib2	13	11	2	
IIa1	13	7	6	
IIa2	6	5	1	
Tumor size (cm)				0.019*
≤4	113	63	50	
>4	19	16	3	
Pathologic types				0.536
Squamous cell carcinoma	106	63	43	
Adenocarcinoma	19	13	6	
Adenosquamous carcinoma	7	3	4	
Differentiation				0.714
Well	6	3	3	
Moderate	63	40	23	
Poor	63	36	27	
Stromal invasion				0.372
<1/2	76	43	33	
≥1/2	56	36	20	
LVSI				0.134
Positive	17	13	4	
Negative	115	66	49	
LNM				0.015*
Positive	26	21	5	
Negative	106	58	48	
Vaginal invasion				0.516
Positive	2	2	0	
Negative	130	77	53	
Parametrial invasion				0.516
Positive	2	2	0	
Negative	130	77	53	

*χ*^2^-test. *P < 0.05.

*FIGO* the International Federation of Gynecology and Obstetrics, *LVSI* lymphovascular space invasion; *LNM* lymph node metastasis.

**Table 2:** Multivariate Cox′s proportional hazards model analysis of disease-free survival and overall survival.

Variables	Overall survival		Disease-free survival	
	HR (95% CI)	*P-*value	HR (95% CI)	*P-*value
Tumor size (cm)		0.013*		0.006**
≤4 (ref)	1		1	
>4	3.327 (1.284–8.621)		3.241 (1.398–7.509)	
LVSI		0.005**		0.003**
Negative (ref)	1		1	
Positive	3.807 (1.495–9.695)		3.805 (1.590–9.104)	
LNM		0.025*		0.016*
Negative (ref)	1		1	
Positive	2.812 (1.136–6.959)		2.764 (1.205–6.340)	
PTPRM		0.038*		0.018*
Low (ref)	1		1	
High	2.517 (1.091–21.439)		4.403 (1.286–15.075)	

*HR* hazard ratio, *CI* confidence interval, *LNM* lymph node metastasis, *LVSI* lymphovascular space invasion, *ref* reference.

For the stepwise multivariate analysis, forward LR method was used to select significant variables. Variables entered for multivariate analysis were the following: tumor size, FIGO stage, LNM, LVSI, PTPRM. *P < 0.05, **P < 0.01, ***P < 0.001.

### Downregulation PTPRM inhibits CCa proliferation, invasion, EMT, and lymphangiogenesis in vitro

**Fig. 3:**
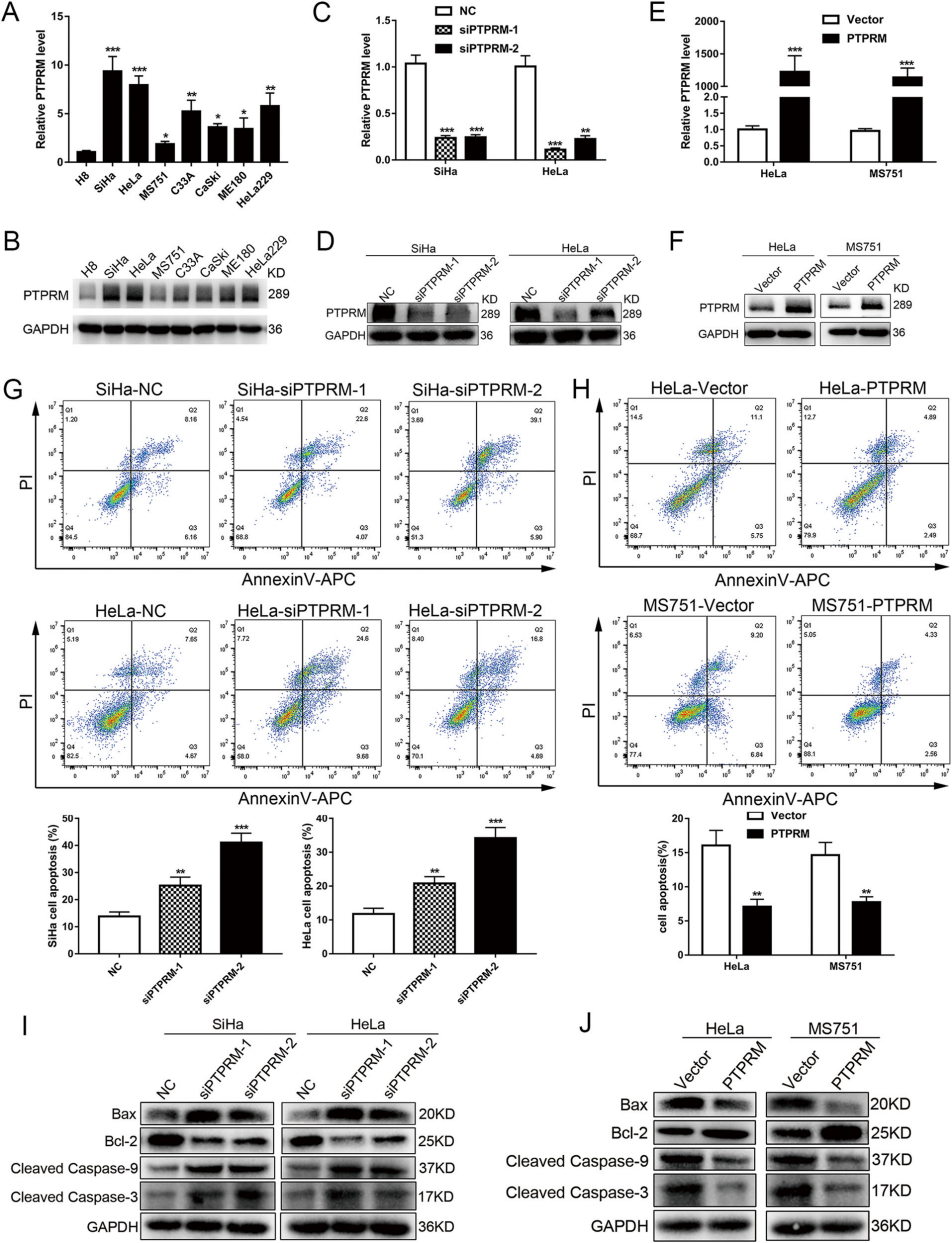
PTPRM inhibits cell apoptosis in vitro. **a**, **b** PTPRM mRNA and protein expression level in 7 CCa cell lines and normal cervix derived cell line H8. **c**–**f** qRT-PCR and Western blot confirmed the efficiency of PTPRM knockdown and overexpression. **g, h** Flow cytometry analysis for Annexin V-APC and PI staining in CCa cells transfected with siRNAs or plasmids for 72 h. **i**, **j** Bax, Bcl-2, cleaved caspase-9 and cleaved caspase-3 expression after PTPRM knockdown or overexpression *, ** or ***: significantly different from the corresponding control, *P* < 0.05, *P* < 0.01 or *P* < 0.001, respectively.

In order to explore the function of PTPRM in CCa, further investigation was conducted with a series of functional assays. At first, we tested the expression level of PTPRM in seven CCa cell lines and normal cervix derived cell line H8 and found that PTPRM expression was up-regulated in CCa cell lines compared with H8 in mRNA and protein levels (Fig. 3a, b). Then we chose the SiHa and HeLa cell lines to knockdown PTPRM, whereas HeLa and MS751 cell lines were selected to overexpress PTPRM. The efficiency of RNA interference and overexpression was confirmed by qRT-PCR and Western blot (Fig. 3c–f). Then the cell apoptosis assay was performed and the results showed that PTPRM knockdown significantly increased cell apoptosis rate of CCa cells, while overexpression of PTPRM decreased cell apoptosis (Fig. 3g, h). Moreover, we found that PTPRM knockdown could increase the expression of Bax, cleaved caspase-9 and cleaved caspase-3 and decrease Bcl-2 expression in SiHa and HeLa cells (Fig. 3i), whereas PTPRM overexpression had the opposite effect on the apoptosis markers expression mentioned above in HeLa and MS751 cells (Fig. 3j). These results indicate that PTPRM could exert anti-apoptosis role in CCa cells.

**Fig. 4:**
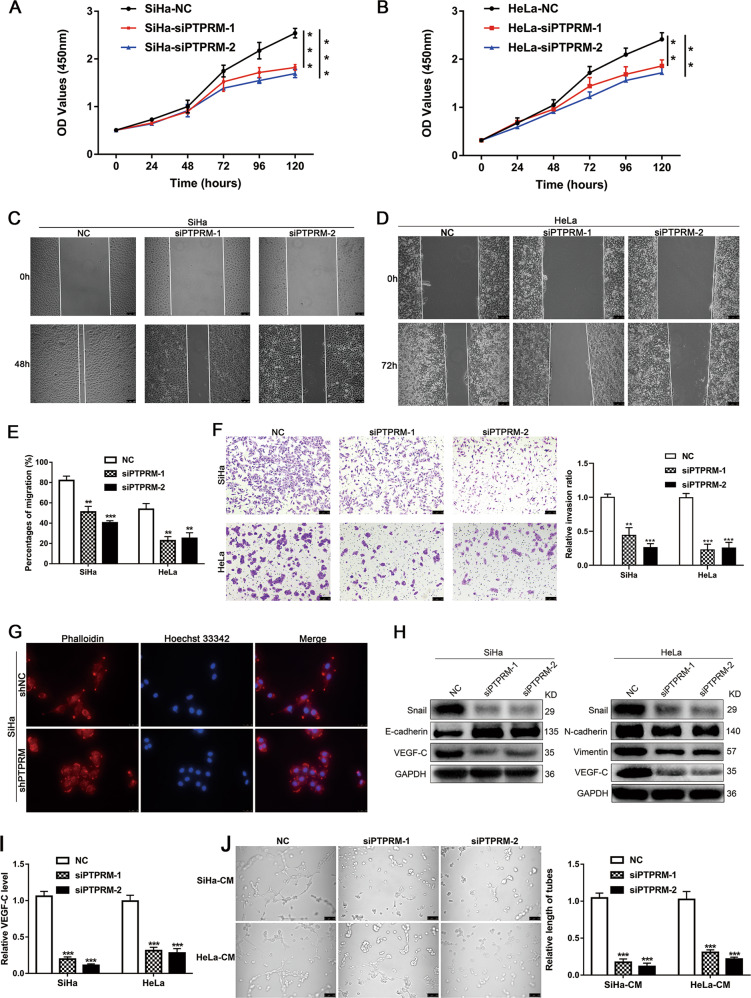
Downregulation PTPRM inhibits CCa proliferation, invasion, EMT, and lymphangiogenesis in vitro. **a**, **b** CCK8 assays revealed that the proliferation ability of SiHa and HeLa cells was significantly inhibited in the PTPRM knockdown cells. **c**–**e** Wound-healing assays were performed to investigate PTPRM knockdown suppressed migration of cervical cancer cells. Original magnification: ×100. **f** Transwell assays showed PTPRM knockdown impeded invasion of cervical cancer cells. Original magnification: ×100. **g** The morphology change of SiHa cells shown by TRITC phalloidin staining after PTPRM knockdown. Original magnification: ×400. **h** Western blot analysis of Snail, E-cadherin, N-cadherin, Vimentin and VEGF-C expression level in CCa cells of NC group and PTPRM knockdown group. **i** qRT-PCR confirmed VEGF-C expression level after PTPRM knockdown in CCa cells. **j** HLECs tube formation assay demonstrated the effect of PTPRM on lymphangiogenesis. Original magnification: ×200. Average length of tube formation was quantified. *, ** or ***: significantly different from the corresponding control, *P* < 0.05, *P* < 0.01 or *P* < 0.001, respectively.

Next, CCK8 assay indicated that PTPRM knockdown markedly decreased the proliferation of SiHa and HeLa cells (Fig. 4a, b). In addition, wound-healing and transwell assays were performed and we found that PTPRM knockdown decreased the migration and invasion ability of SiHa and HeLa cells (Fig. 4c–f). Since PTPRM could influence cell migration and invasion, we further examined whether PTPRM can induce EMT of cancer cells. We investigated the correlation of PTPRM and EMT markers in our CCa tissues cohort and the results showed that PTPRM was significantly positively correlated with Snail (*r* = 0.718, *P* < 0.001), E-cadherin (*r* = −0.579, *P* < 0.001) and Vimentin (*r* = 0.504, *P* < 0.01) (Supplementary Fig. S1a–c). In TCGA database, PTPRM is positively correlated with Vimentin, Snail, ZEB1, and ZEB2 (Supplementary Fig. S1d–f). Moreover, we found that SiHa cells changed from a strip shape to a rounded one after PTPRM knockdown through TRITC phalloidin fluorescent staining (Fig. 4g). Furthermore, knockdown of PTPRM could significantly increase the expression of E-cadherin and decrease the expression of Snail, N-cadherin and Vimentin in CCa cells (Fig. 4h; Supplementary Fig. S1h, i), suggesting that PTPRM knockdown inhibited EMT of CCa cells. In addition, our results showed that VEGF-C, a key factor involved in lymphangiogenesis, was decreased in the PTPRM knockdown cells at both the mRNA and protein levels (Fig. 4h, i). Furthermore, we investigated the effect of PTPRM on the tube formation of human lymphatic endothelial cells (HLECs), which is important for LNM. Compared with corresponding control groups, the culture medium supernatant of PTPRM knockdown cells significantly suppressed HLECs tube formation (Fig. 4j). Taken together, these results demonstrate that knockdown of PTPRM could suppress proliferation, invasion, EMT, and lymphangiogenesis in CCa.

### Overexpression PTPRM promotes CCa proliferation, invasion, EMT, and lymphangiogenesis in vitro

**Fig. 5:**
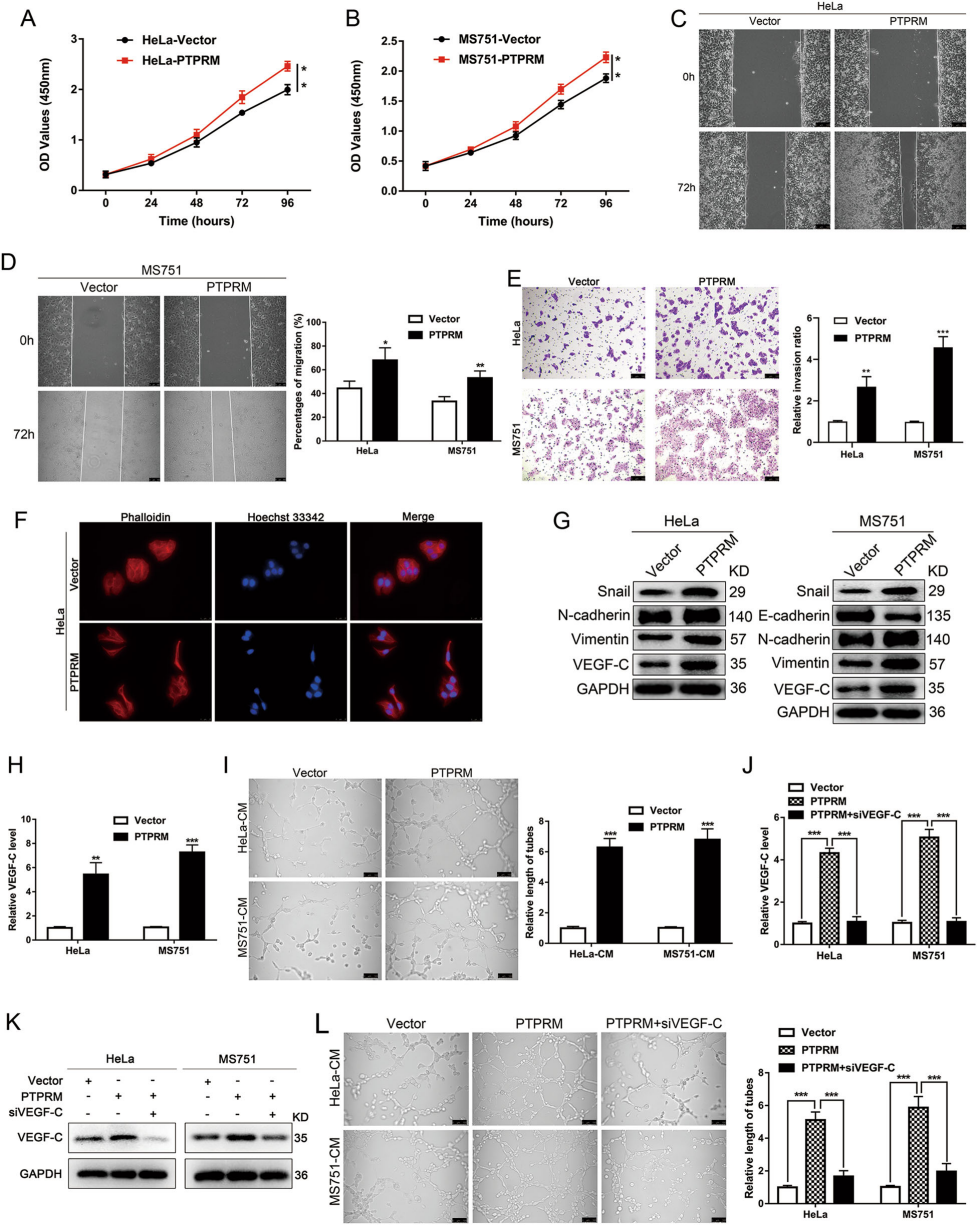
PTPRM overexpression promotes CCa proliferation, invasion, EMT, and lymphangiogenesis in vitro. **a**, **b** CCK8 assay showed PTPRM overexpression promoted the proliferation of CCa cells. **c**–**e** Wound healing and transwell assays indicated that PTPRM overexpression increased the migration and invasion ability of CCa cells. Original magnification: ×100. **f** The morphology change of HeLa cells shown by TRITC phalloidin staining after PTPRM overexpression. Original magnification: ×400. **g** Snail, E-cadherin, N-cadherin, Vimentin and VEGF-C expression level were confirmed by Western blot in PTPRM overexpression cells. **h** qRT-PCR confirmed VEGF-C expression level after PTPRM expression in CCa cells. **i** PTPRM overexpression promoted HLECs tube formation. **j**–**k** VEGF-C mRNA and protein levels after PTPRM overexpressed cells were treated with siVEGF-C. **l** VEGF-C knockdown abrogated PTPRM overexpression induced lymphangiogenesis. Total length of tube formation was quantified. Original magnification: ×200. *, ** or ***: significantly different from the corresponding control, *P* < 0.05, *P* < 0.01 or *P* < 0.001, respectively.

We overexpressed PTPRM in HeLa and MS751 cells and CCK8 assay results suggested that PTPRM overexpression promoted CCa cells proliferation (Fig. 5a, b). Besides, wound-healing and transwell experiments indicated that PTPRM overexpression increased migration and invasion capabilities of CCa cells (Fig. 5c–e). Moreover, overexpression of PTPRM changed HeLa cells from a rounded shape to an elongated one (Fig. 5f), and significantly decreased the E-cadherin in MS751 cells and increased Snail, N-cadherin and Vimentin expression in HeLa and MS751 cells (Fig. 5g), suggesting that PTPRM could induce CCa cells EMT. Moreover, we found increased VEGF-C expression after overexpression PTPRM both in mRNA and protein levels in CCa cells (Fig. 5g, h), and the culture medium supernatant of PTPRM overexpressed cells could promote HLECs tube formation (Fig. 5i). Furthermore, knockdown VEGF-C in overexpressed PTPRM cells could abrogate the promoting lymphangiogenesis effect of PTPRM (Fig. 5j–l), which indicated that PTPRM could promote lymphangiogenesis in a VEGF-C dependent manner. Taken together, these results suggest that PTPRM overexpression could promote CCa proliferation, invasion, EMT, and lymphangiogenesis in vitro.

### Knockdown PTPRM reduces tumor growth, lymphangiogenesis and LNM in vivo

**Fig. 6:**
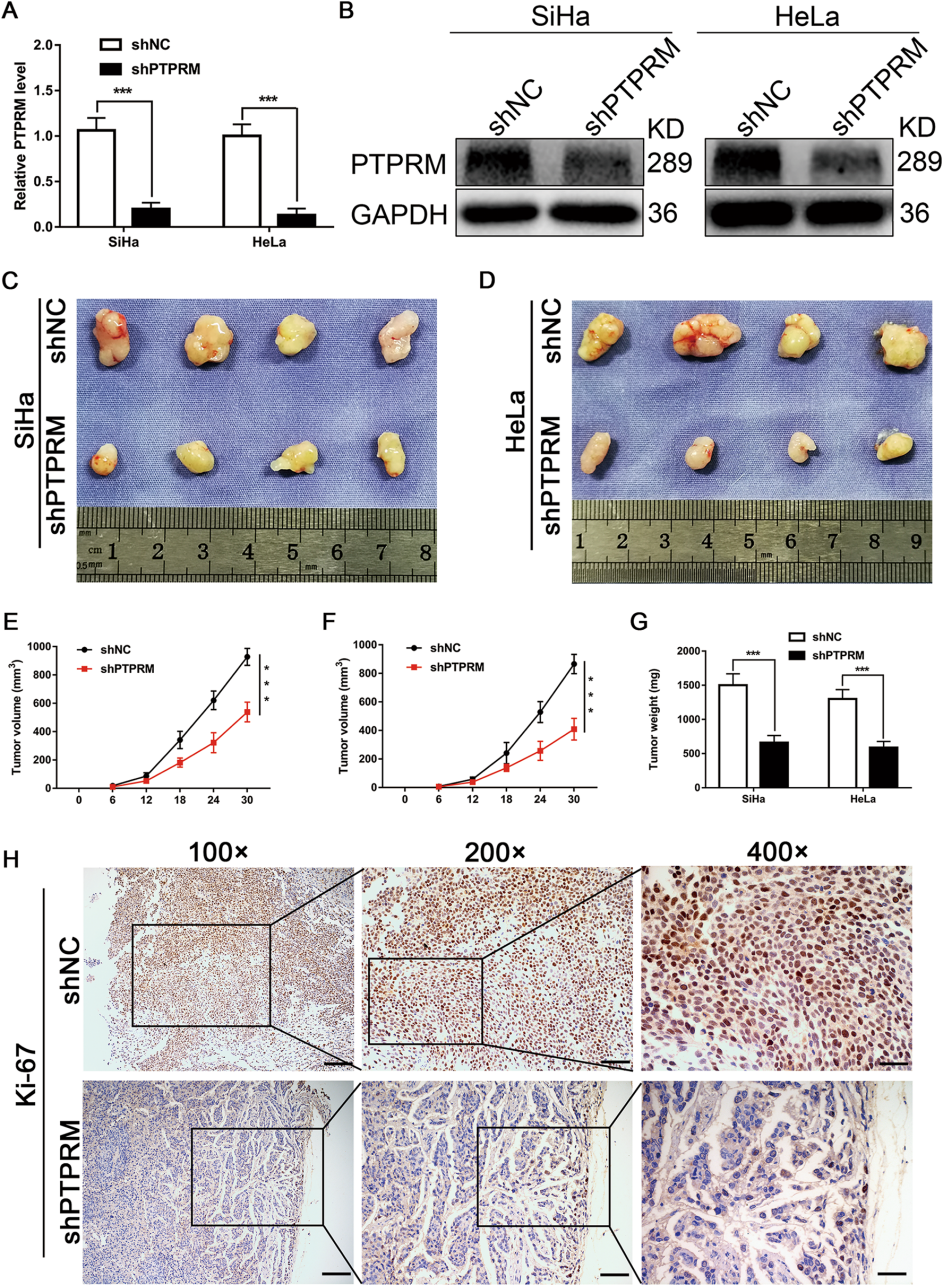
PTPRM promotes tumor growth of CCa in vivo. **a**, **b** qRT-PCR and Western blot were used to confirm the efficiency of transduction shPTPRM in SiHa and HeLa cells. **c**, **d** The representative pictures of subcutaneous tumors from nude mice injected cells for 30 days (n = 10 per group). **e**–**g** Subcutaneous tumor volume and weight of mice in different treatment groups were displayed. **h** The Ki-67 expression levels of subcutaneous tumors from shNC and shPTPRM groups mice. Original magnification, ×100, ×200, ×400. *, ** or ***: significantly different from the corresponding control, *P* < 0.05, *P* < 0.01 or *P* < 0.001, respectively.

**Fig. 7:**
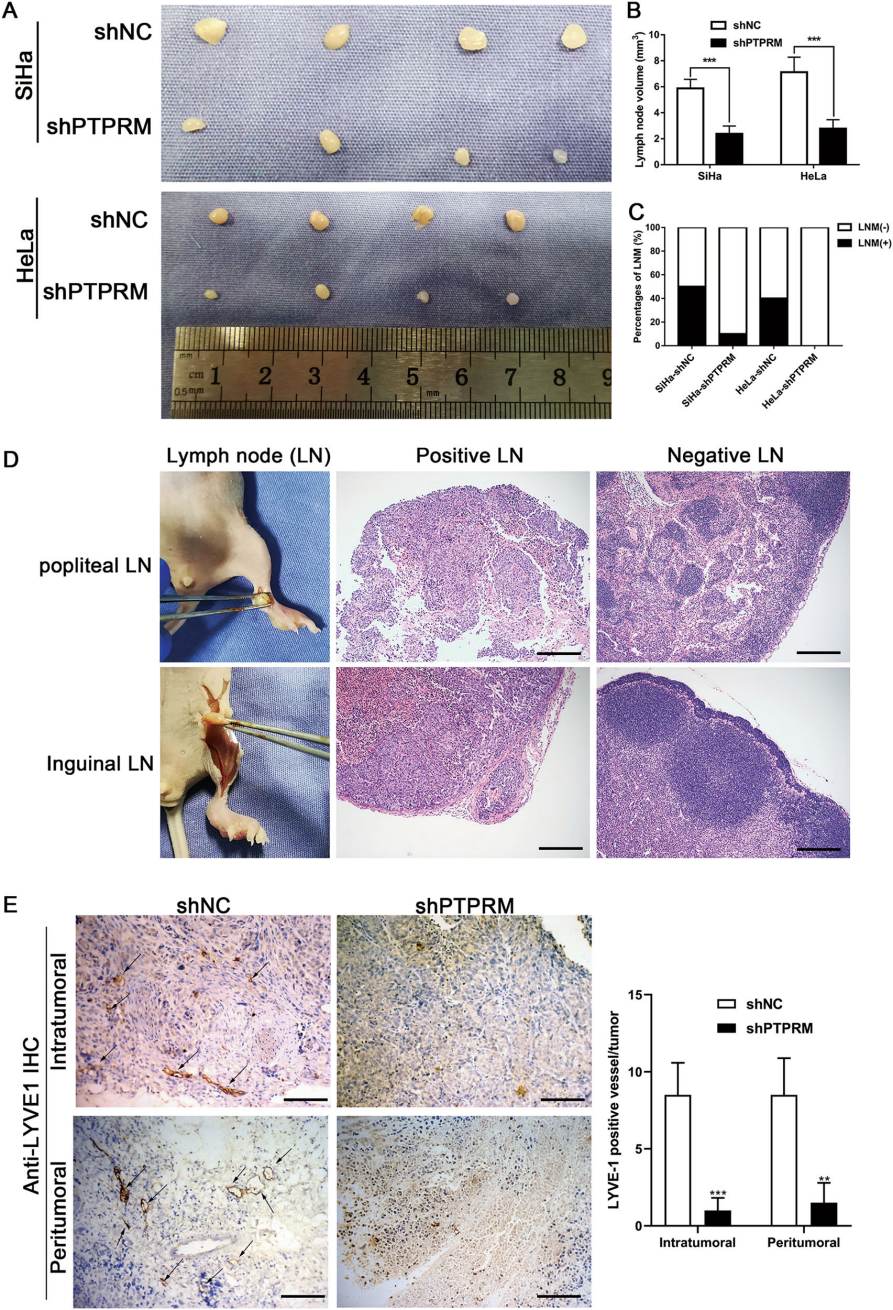
PTPRM knockdown suppresses the lymphangiogenesis and LNM of CCa in vivo. **a** The representative pictures of popliteal lymph nodes (*n* = 10 per group). **b**, **c** Histogram analysis of the average popliteal lymph nodes volume and the rate of LNM in different treatment groups. **d** The representative pictures of H-E staining of positive and negative metastatic popliteal lymph nodes and inguinal lymph nodes. Original magnification: ×100. **e** Representative IHC pictures of intratumoral and peritumoral lymphatic vessels in the primary tumors resected from footpads of nude mice stained by anti-LYVE-1 in the mice bearing PTPRM-silenced cells and control group (left panel), quantification of LYVE-1 positive vessels in primary tumors (right panel). Original magnification: ×200. *, ** or ***: significantly different from the corresponding control, *P* < 0.05, *P* < 0.01 or *P* < 0.001, respectively.

The effects of PTPRM on tumor growth and LNM were further confirmed by animal experiments. PTPRM was stable knockdown in SiHa and HeLa cells by transduction of lenti-virus packaged shPTPRM and the efficiency of knockdown was confirmed both in mRNA and protein levels (Fig. 6a, b). Subcutaneous xenograft tumor model and lymphatic metastatic model in the female BALB/c nude mice were adopted. After continuous monitoring the tumor volume for 30 days, we found that tumors volume in shPTPRM group was less than shNC groups (Fig. 6c–f). Moreover, tumor weight of shPTPRM group was also less than shNC group (Fig. 6g). Furthermore, the proliferation marker Ki-67 was lower expressed in shPTPRM group tumors than shNC group tumors (Fig. 6h). We further examined whether PPTRM knockdown inhibits lymphangiogenesis and LNM in the animal model. After injecting tumor cells into the foot pads of nude mice for 30 days, popliteal and inguinal lymph nodes were removed (Fig. 7a). We found that the volume of popliteal lymph nodes was smaller in the shPTPRM group than that in the control group (Fig. 7b). Moreover, our results showed that the rate of LNM declined in the mice transplanted with PTPRM knockdown cells (Fig. 7c). The status of LNM was validated by H&E staining (Fig. 7d). Importantly, the quantity of intratumoral and peritumoral lymphatic vessels in the primary tumors resected from footpads of nude mice, which were assessed using an antibody to a lymphatic marker, LYVE-1, were dramatically decreased in the mice bearing PTPRM-silenced cells (Fig. 7e), indicating that PTPRM knockdown could repress lymphangiogenesis in vivo. These data indicate that PTPRM ablation could repress tumor growth, lymphangiogenesis and LNM of CCa in vivo.

### PTPRM promotes CCa proliferation and metastasis via Src-AKT signaling pathway

**Fig. 8:**
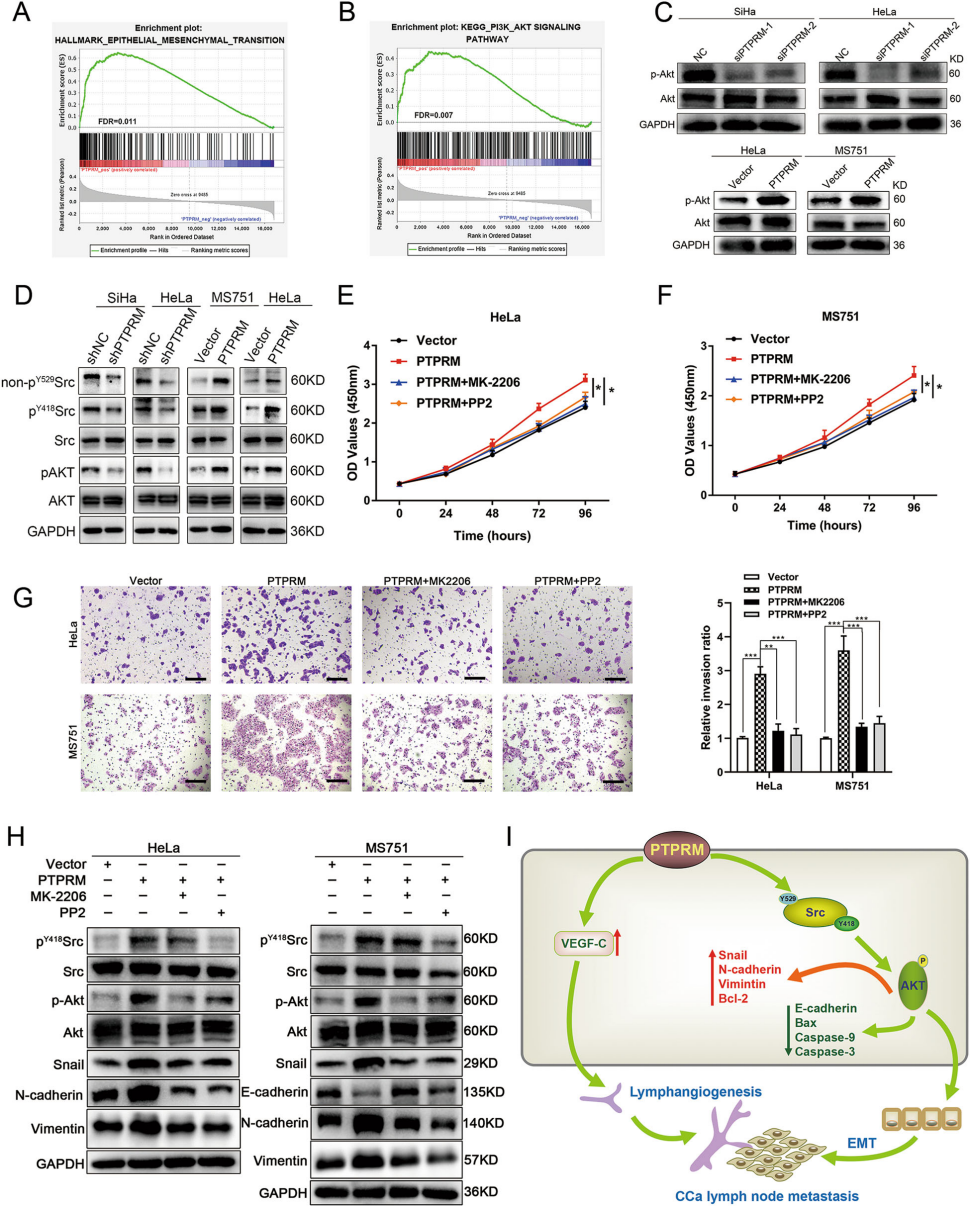
PTPRM promotes CCa proliferation and metastasis through Src-AKT signaling pathway. **a**, **b** GSEA showed PTPRM was positively correlated with AKT and EMT pathway. **c** Western blot analysis revealed p-AKT level in CCa cells with PTPRM knockdown or overexpression. **d** Western blot analysis revealed non-p^Y529^Src, p^Y418^Src, and p-AKT levels in CCa cells with PTPRM knockdown or overexpression. **e**, **f** CCK8 assay suggested that PP2 and MK-2206 abrogated the promoting effect of PTPRM upregulation on cell proliferation ability in HeLa and MS751 cells. **g** Transwell assay demonstrated that PP2 and MK-2206 alleviated the increased invasion capability due to PTPRM overexpression in HeLa and MS751 cells. **h** Western blot analysis showed that p^Y418^Src, p-AKT, Snail, E-cadherin, N-cadherin, Vimentin expression levels were partly rescued by PP2 and MK-2206 treatment in CCa cells. **i** Schematic illustration showing the proposed mechanism by which PTPRM promotes CCa lymph node metastasis. *, ** or ***: significantly different from the corresponding control, *P* < 0.05, *P* < 0.01 or *P* < 0.001, respectively.

In order to explore the potential mechanism of PTPRM promoting cervical cancer cell proliferation and metastasis, gene set enrichment analysis (GSEA) was used and the results demonstrated that PI3K/AKT and EMT pathway were correlated to the PTPRM expression (Fig. 8a, b). Cumulative results showed that the dysregulation of the PI3K/AKT signaling pathway was common in human cancers, including ovarian, breast, prostate and cervical cancer^15–19^. Meanwhile, aberrant activation of the PI3K/AKT signaling pathway could contribute to the CCa cell proliferation and angiogenesis^20–22^. Therefore, we hypothesized that PTPRM could promote CCa progression via regulating AKT signaling pathway. We found that PTPRM overexpression significantly increased, while PTPRM knockdown reduced the expression of p-AKT in CCa cells (Fig. 8c, d). These results suggested that the AKT signaling pathway participated in the PTPRM-induced CCa progression. Subsequently, we explored the mechanism by which PTPRM activated AKT signaling pathway. It has been reported that protein tyrosine phosphatase PTPRJ, also a member of PTP family, could dephosphorylates the c-Src inhibitory tyrosine phosphorylation site (Tyr 529), thereby increasing Src and AKT pathway activity^23–25^. Meanwhile, PTPRJ could promote sprouting angiogenesis through Src-AKT pathway in endothelial cells^25^, thus we hypothesized that PTPRM activated AKT signaling via increasing Src activity. Our results showed that PTPRM overexpression could increase the dephosphorylation of Src-Y529 (non-p^Y529^Src), p^Y418^Src and p-AKT expression in CCa cells, whereas PTPRM knockdown had the converse effect, suggesting that PTPRM could enhance Src tyrosine kinase activity through dephosphorylating the c-Src Tyr-529 site, thereby inducing c-Src self-activation and consequent AKT activation (Fig. 8d). Meanwhile, we found that PP2, a Src pathway inhibitor, could reverse p-AKT expression which was augmented by PTPRM overexpression in CCa cells, indicating that PTPRM activated AKT signaling in a Src pathway dependent manner (Fig. 8h).

To explore whether PTPRM promoted CCa cells proliferation and aggressiveness via Src-AKT pathway, we performed rescue experiments. Our results demonstrated that increased cell proliferation and invasion abilities caused by PTPRM upregulation could be abrogated by both Src pathway inhibitor PP2 and AKT pathway inhibitor MK-2206 in MS751 and HeLa cells (Fig. 8e–g). Moreover, we found that the expression level of E-cadherin was partially increased, whereas the expression level of Snail, N-cadherin and Vimentin were partly decreased in PTPRM overexpressed cells by PP2 and MK-2206 treatment (Fig. 8h). Collectively, these results indicate that PTPRM could promote CCa proliferation and metastasis through Src-AKT signaling pathway.

## Discussion

LNM is a key problem that significantly influence the clinical prognosis of patients with CCa^3–5^. Focusing on the crucial molecules which could exert pro-metastatic functions may help clinicians to understand the molecular mechanism of LNM and develop clinical therapeutic strategies. However, the precise mechanism is largely unknown. Herein, we identified PTPRM from TCGA dataset, which plays an important role in the progression of CCa. Subsequently, we demonstrated that PTPRM was upregulated in cervical cancer with LNM, correlated with poor prognosis and LNM. Through gain and loss of function approaches, we found that PTPRM promoted CCa cells proliferation, migration and lymphangiogenesis. Furthermore, PTPRM promoted EMT via the activation of Src-AKT signaling pathway and induced lymphangiogenesis in a VEGF-C dependent manner, resulting in LNM of CCa. Importantly, knockdown of PTPRM dramatically reduced lymphangiogenesis and LNM in vivo, suggesting that PTPRM plays a crucial role in the LNM of CCa and may represent a potential molecular target for clinical intervention in patients of CCa with LNM.

It has been reported that PTPs play an important role in regulating many biologic processes in cancer development^8^. In this study, we found that PTPRM was upregulated in CCa tissue with LNM, and PTPRM was positively associated with LNM in patients with CCa. Moreover, our results showed that PTPRM could promote EMT, lymphangiogenesis, and LNM of CCa. Similar to our study, Wang Y et al. and Gebbink M.F et al. demonstrated that PTPRM plays oncogenic role in lung cancer^14,26^. However, some studies indicated that PTPRM was negatively correlated with the progression of colorectal adenoma-carcinoma, small intestinal neuroendocrine tumors and breast cancer^11–13^. To our knowledge, there has been few functional studies of PTPRM in cancer cells to date, and published literature on PTPRM mostly came from the bioinformatic analysis aspects lacking of experiments verification. For this situation, we speculate that whether PTPs function as oncogenes or tumor suppressor genes is cellular context dependent and the newly discovered role of PTPRM in promoting the progression of CCa may be due to the tumor heterogeneity.

It is well-established that EMT plays important roles in cancer progression, especially in tumor metastasis^27–30^. Our previous studies have confirmed the close relationship between EMT and CCa metastasis. LncRNA LNMICC could promote EMT of CCa by reprogramming fatty acid metabolism and then facilitate lymphatic metastasis^31^. TRIM62 could suppress CCa metastasis via c-Jun/Slug signaling mediated EMT^32^. Herein, we showed that downregulation of PTPRM could inhibit EMT and overexpression of PTPRM promoted EMT of CCa cells. Further mechanistic investigation revealed that PTPRM promoted EMT via Src-AKT signaling pathway, providing a novel mechanism underlying CCa metastasis.

Another important finding in our study is PTPRM could promote the expression of VEGF-C in cervical cancer cells, resulting in lymphangiogenesis. Lymphangiogenesis, a critical early metastatic event, is important for LNM and is a strong prognostic factor of survival for CCa patients^7,33,34^. Accumulating evidences have demonstrated that inhibition of lymphangiogenesis could prevent LNM in vivo and prolong the survival time of animals in many cancer types^35–37^. Herein, our results indicated that downregulation of PTPRM prevented lymphangiogenesis and inhibited the incidence of LNM in CCa in vitro and in vivo, suggesting that PTPRM may serve as a potential target for treatment in CCa. VEGF-C, a lymphatic vessel specific growth factor, has been shown to disrupt the endothelial lymphatic barrier and enhance lymph node metastasis of cancer cells^38–41^. Moreover, several studies showed that the inhibition of VEGF-C could reduce the rate of LNM and suppress the dissemination of cancer cells from lymph node to distant organs^42,43^. In this study, we found that overexpression of PTPRM could increase the VEGF-C expression level in CCa cells and facilitate the formation of new lymphatic vessels. Besides, PTPRM knockdown could repress lymphangiogenesis in vivo. Moreover, depletion of VEGF-C using siRNAs in PTPRM-overexpressed CCa cells indicated promising antitumor effects via inhibiting lymphangiogenesis and LNM.

Our results show that PTPRM plays dual promoting lymphatic metastasis roles including promotion of lymphangiogenesis and enhancement of invasiveness, providing the mechanistic and translational insight into the lymphatic metastasis of CCa. As the RNA interference technique and monoclonal antibody clinical therapeutic application, nanovesicle delivery system packaged with siRNAs or other targeting PTPRM drugs might exert potential anti-tumor role in CCa treatment in the future.

In summary, our findings provide solid evidences that PTPRM upregulation is clinically and functionally relevant to LNM of CCa through VEGF-C dependent lymphangiogenesis and Src-AKT signaling pathway mediated EMT. Moreover, PTPRM could serve as a novel prognostic factor in CCa. Our study not only brings novel insight into the molecular mechanism underlying LNM of CCa, but also develops a new potential therapeutic target for CCa patients with LNM.

## Materials and methods

### Cell lines and cell culture

Seven CCa cell lines, including Siha, HeLa, Caski, MS751, ME180, C33A and HeLa229, and normal cervix derived cell line H8 were purchased from ATCC and cultured in a humidified atmosphere with 5% CO_2_ at 37 °C. Human lymphatic endothelial cells (HLECs) were obtained from ScienCell Research Laboratories and maintained in the recommended endothelial cell medium (ScienCell, CA). All cell lines were cultured in complete medium with 10% FBS (Gibco, USA) as previously described^31^.

### Tissue specimens

We obtained 132 paraffin-embedded CCa (IA2 to IIA) tissues from 2006 to 2013 from the First Affiliated Hospital of Sun Yat-sen University (Guangzhou, China). Normal cervix tissues were obtained from patients who underwent hysterectomy under non-malignant conditions. Another 12 LACC tissues (IB2 and IIA2) and 24 ECC tissues were collected for Western blot and qRT-PCR. None of the patients were exposed to chemical or radical therapy before surgery. All patients signed the informed consent. Specimens used in this study were approved by the Ethical Review Committee of the First Affiliated Hospital of Sun Yat-sen University.

### Quantitative real-time PCR (qRT-PCR), immunohistochemistry (IHC) and Western blot

qRT-PCR, IHC and Western blot were performed as previously described^31,32^. All the primers were synthesized by GENEWIZ (Suzhou, China) and primer sequences were listed in Supplementary Table S1. Primary antibodies used in this study were listed in Supplementary Table S2. For IHC statistical analysis, the immunostaining scores (ranging from 0 to 6) were evaluated and a cut-off 4 was determined^44^. The results of IHC were determined by specialized pathologists.

### Vector construction and cell transfection

siRNAs of PTPRM and negative control (NC) were designed and synthesized by GenePharma (Suzhou, China) and were transfected into SiHa and HeLa cells using Lipofectamine RNAiMAX (Invitrogen, USA) following the manufacturer′s protocol. siRNA sequences were showed in Supplementary Table S1. To overexpress PTPRM, the pCDH-V5-His/Puro-PTPRM plasmid was synthesized by Sangon Biotech (Shanghai, China) and then transfected into MS751 and HeLa cells using X-tremeGENE HP DNA transfection reagent (Sigma-Aldrich, USA). Lentivirus packaged shPTPRM and shNC were purchased from GenePharma and the stably transfected cells were screened by puromycin (Sigma-Aldrich, 3 mg/ml) for at least 5 days.

### CCK8 assay and apoptosis assay

CCK8 and apoptosis assay were performed as previously described^31^. CCK8 assay kit (Dojindo, Japan) and apoptosis Detection Kit (Keygen Biotech, China) were used according to the manufacturer′s instructions. After cells were transfected with siRNAs or vectors for 72 h, cell apoptotic rate was measured by a flow cytometer (Beckman Coulter) and analyzed by FlowJo software. Each assay was repeated three times.

### Cytoskeleton fluorescent staining

With the purpose of cytoskeleton analysis, TRITC phalloidin staining was performed to mark F-actin in CCa cells. Cells which grown on glass coverslips were washed twice with preheated (37 °C) PBS for 5 min each time and fixed for 10 min in 4% formaldehyde dissolved in PBS. Then cells were permeabilized with 0.5% Triton X-100 in PBS for 5 min and blocked with 1% bovine serum albumin (BSA) in PBS for 15 min. F-actin was stained with TRITC phalloidin (Solarbio, Beijing, China) in PBS containing 1% BSA for 40 min at room temperature as the manufacturer′s protocol. Wash several times with PBS to remove unbound TRITC phalloidin. Finally, cells were incubated with Hoechst 33342 (Beyotime, China) and then washed three times with PBS. The cells were imaged by an inverted fluorescence microscope Leica DMI8.

### Wound healing and cell invasion assays

Cells were seeded in a 6-well plate and then transfected with siPTPRM or PTPRM plasmid until reaching 100% confluence. Then the cells in culture dish were preincubated with Mitomycin C (10 μg/ml) for 1 h at 37 °C to suppress proliferation. For wound healing assay, a scratch presenting a wound was set and then cells were cultured for 72 h. For cell invasion assay, cells were seeded into the upper chamber (Corning, USA) which was pre-coated with 15% matrigel (BD, USA), while the lower chamber was filled with 500 μl medium containing 10% FBS. After 48 h, the cells on the lower surface of the chamber were fixed and then stained. The numbers of invaded cells were counted under microscope.

### HLECs tube formation assay

FBS-free culture medium supernatant obtained from the co-culture of cancer cells was concentrated ten-fold using ultrafiltration spin columns (Millipore, USA). HLECs were seeded into 96-well plates (pre-coated with matrigel) containing concentrated culture medium and incubated for 6 h. The lymphatic tubes were photographed using Leica DMI8 microscope and quantified by measuring the length of the completed tubule structures.

### Xenograft model

Female BALB/c nude mice (4–6 weeks of age, 18–20 g) were raised under SPF conditions in the Sun Yat-sen University Animal Center and randomly divided into 4 groups (*n* = 10 per group). For subcutaneous tumor model, stably downregulated PTPRM cancer cells (1 × 10^7^ per mouse) were inoculated into the shoulder of nude mice. Tumor′s volume was calculated according to the formula: length × width^2^ × 0.52^45^. For LNM model, the cells (2 × 10^6^ per mouse) were directly inoculated into the foot pad of mice. All mice were sacrificed at the 30th day after inoculation and all tumors and lymph nodes were removed for further analysis. The number of metastatic foci was counted and diagnosed by specialized pathologists by HE staining. Animal experiments were approved by the Animal Ethical and Welfare Committee of Sun Yat-sen University.

### Statistical analysis

SPSS 20.0 and GraphPad prism 7.0 software were used for statistical analysis. Unpaired Student *t*-test was used to analyze the differences between 2 groups. One-way analysis of variance was used to evaluate the differences among multiple groups. The Kaplan-Meier method was used for overall survival and disease-free survival analysis, and significance was determined by log-rank test. Multivariate Cox regression analyses was performed to evaluate independent prognostic factors of cervical cancer. The *χ*^2^ test and Fisher′s exact test were used to analyze the relationship between PTPRM expression and the clinicopathological characteristics. The data was presented as the mean ± SD. of at least three independent experiments. A value of *P* < 0.05 was regarded as statistically significant.

## Supplementary information

Supplementary Figure 1

Supplementary Figure 2

Supplementary Figure 3

Supplementary Figure Legends

Supplementary Table S1-S3
