# Supplementary figures and images for: High expression of PTPRM predicts poor prognosis and promotes tumor growth and lymph node metastasis in cervical cancer
Source: Cell Death Dis. 2020 Aug 11;11(8):687. doi: 10.1038/s41419-020-02826-x (PMC7443137; doi:10.1038/s41419-020-02826-x)

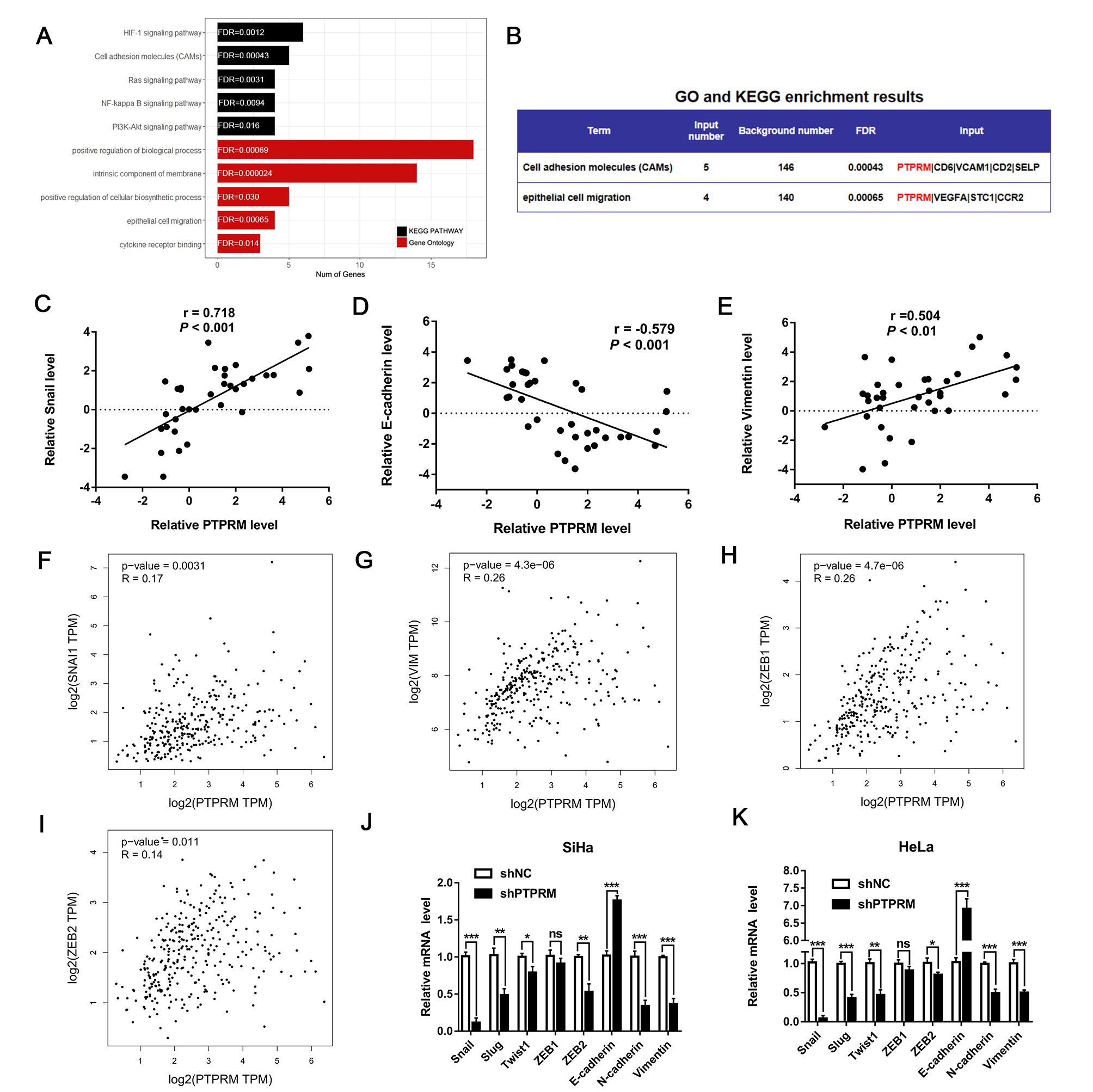

Supplement: Supplementary file 1 — Supplementary Figure 1 [file 41419_2020_2826_MOESM1_ESM.tif]

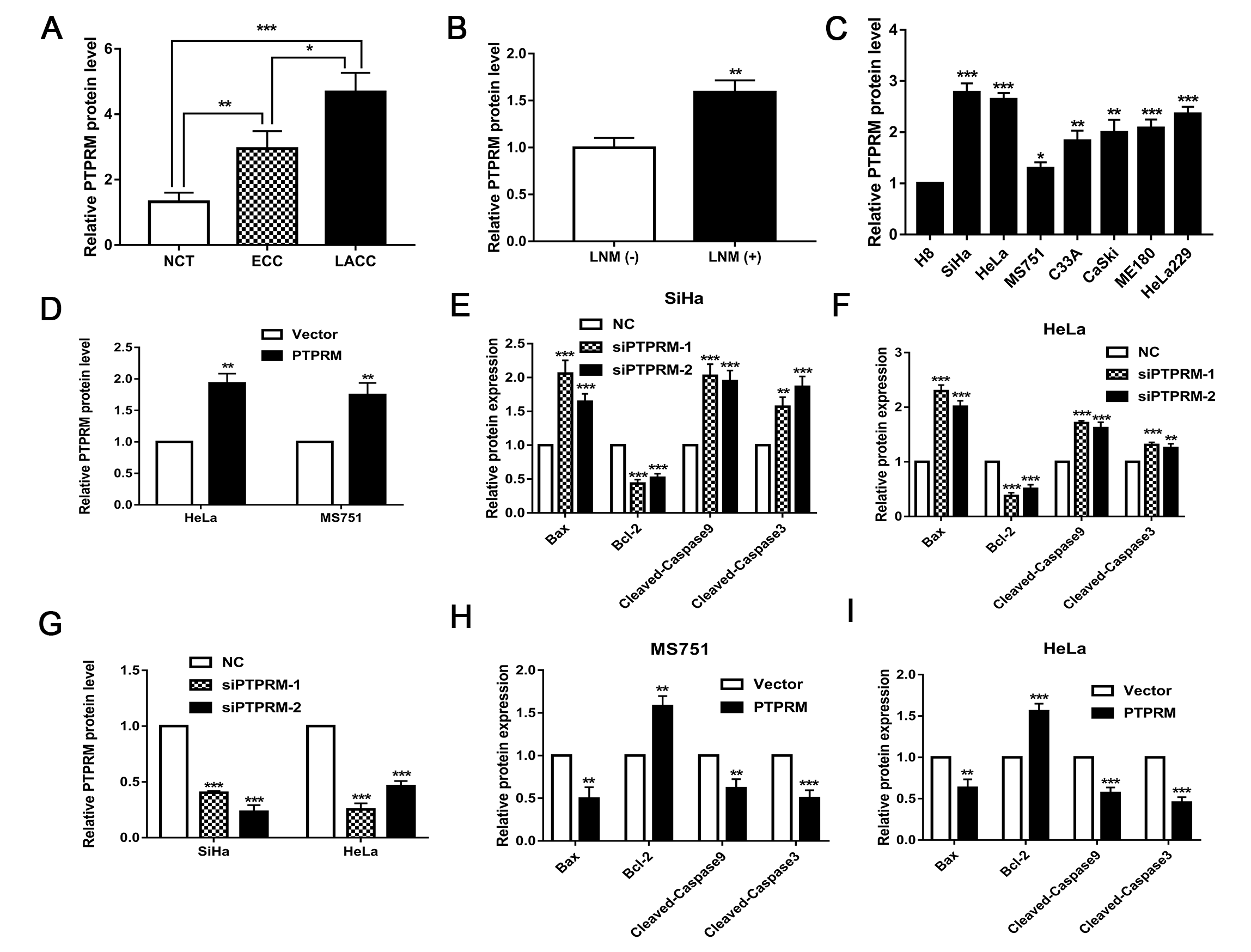

Supplement: Supplementary file 2 — Supplementary Figure 2 [file 41419_2020_2826_MOESM2_ESM.tif]

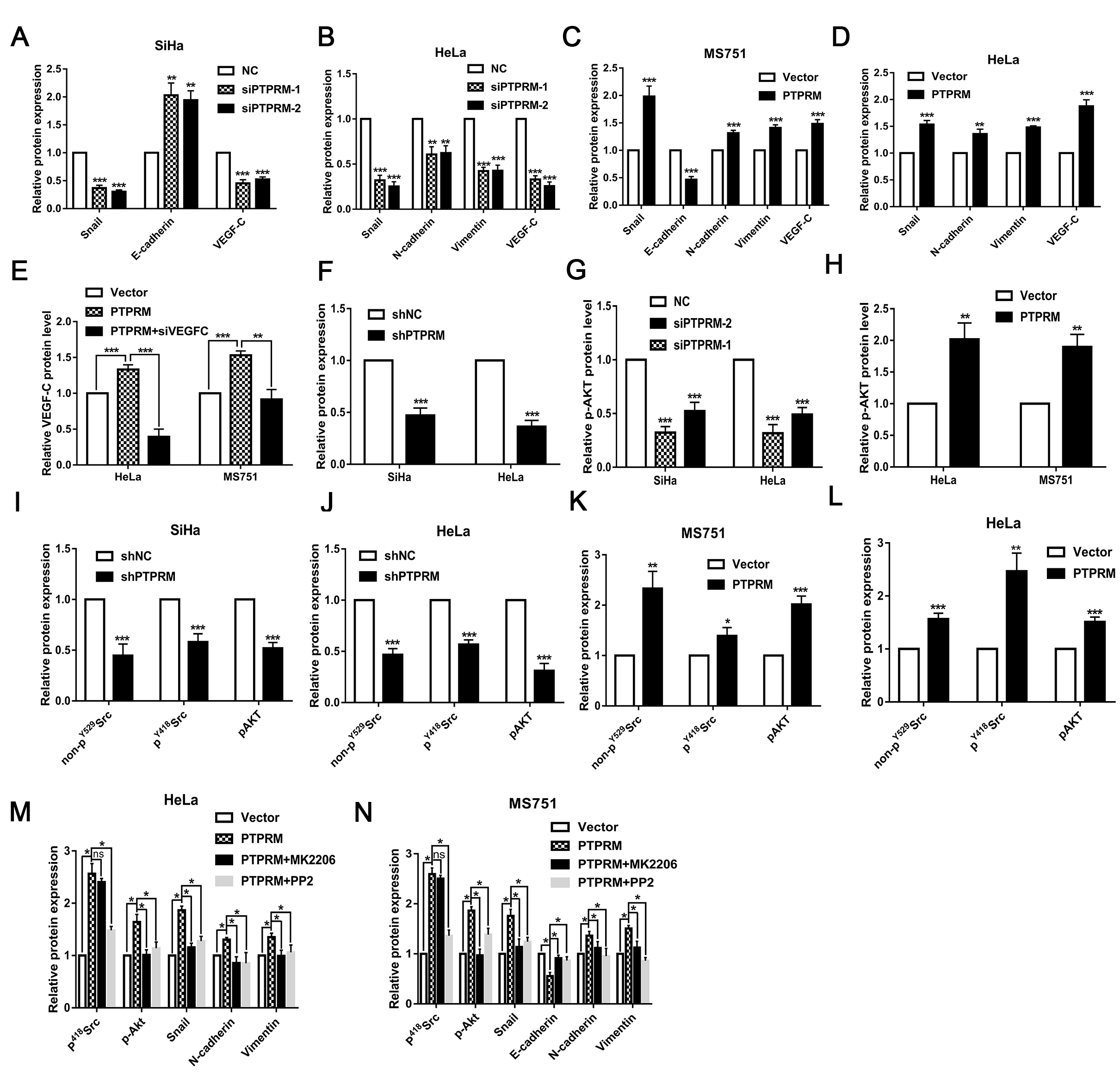

Supplement: Supplementary file 3 — Supplementary Figure 3 [file 41419_2020_2826_MOESM3_ESM.tif]
