# Supplementary material for: High expression of PTPRM predicts poor prognosis and promotes tumor growth and lymph node metastasis in cervical cancer
Source: Cell Death Dis. 2020 Aug 11;11(8):687. doi: 10.1038/s41419-020-02826-x (PMC7443137; doi:10.1038/s41419-020-02826-x)
Supplement: Supplementary file 4 — Supplementary Figure Legends [file 41419_2020_2826_MOESM4_ESM.docx]

Supplementary Figure S1

**Figure legend: a-c** Correlation analysis between PTPRM and Snail, E-cadherin, or Vimentin expression analyzed by qRT-PCR in our CCa tissue cohort (n = 36). **d-f** Correlation analysis between PTPRM and Snail, Vimentin, ZEB1, ZEB2 expression in TCGA CESC dataset. **h, i** Relative mRNA levels of EMT markers and EMT transcript factors after PTPRM knockdown in SiHa and HeLa cells. *, ** or *** represents P < 0.05, P < 0.01 or P < 0.001, respectively.

Supplementary Figure S2

**Figure legend:** Quantification of Western blots in Fig 2-3. *, ** or *** represents P < 0.05, P < 0.01 or P < 0.001, respectively.

Supplementary Figure S3

**Figure legend:** Quantification of Western blots in Fig 4-8. *, ** or *** represents P < 0.05, P < 0.01 or P < 0.001, respectively.
