# Supplementary material for: High expression of PTPRM predicts poor prognosis and promotes tumor growth and lymph node metastasis in cervical cancer
Source: Cell Death Dis. 2020 Aug 11;11(8):687. doi: 10.1038/s41419-020-02826-x (PMC7443137; doi:10.1038/s41419-020-02826-x)
Supplement: Supplementary file 5 — Supplementary Table S1-S3 [file 41419_2020_2826_MOESM5_ESM.docx]

**Table S1 Primers for qRT-PCR and sequences of siRNAs**

| PTPRM primer | Forward: 5′-AAGAGACCATGAGCAGCACC-3′ |
| --- | --- |
|  | Reverse: 5′-ATCACCATCTTCCAGGAGCGA-3′ |
| GAPDH primer | Forward: 5′-CCTTCTCCATGGTGGTGAAGAC-3′ |
|  | Reverse: 5′-AATGAGAAAGCCTCGTCGCA-3′ |
| VEGF-C primer | Forward: 5′-GGCTGGCAACATAACAGAGAA-3′ |
|  | Reverse:5′-CCCCACATCTATACACACCTCC-3′ |
| E-cadherin primer | Forward:5′- ATTCTGATTCTGCTGCTCTTG-3′ |
|  | Reverse:5′- AGTAGTCATAGTCCTGGTCTT-3′ |
| N-cadherin primer | Forward:5′-TCAGGCGTCTGTAGAGGCTT-3′ |
|  | Reverse:5′-ATGCACATCCTTCGATAAGACTG |
| Vimentin primer | Forward:5′-ATTGCCACCTACAGGAAGCT-3′ |
|  | Reverse:5′-GCAGAAAGGCACTTGAAAGC-3′ |
| Snail primer | Forward:5′-GAGGCGGTGGCAGACTAG-3′ |
|  | Reverse:5′-GACACATCGGTCAGACCAG-3′ |
| Slug primer | Forward:5′-CATGCCTGTCATACCACAAC-3′ |
|  | Reverse:5′-GGTGTCAGATGGAGGAGGG-3′ |
| Twist1 primer | Forward:5′-CGGGAGTCCGCAGTCTTA-3′ |
|  | Reverse:5′-TGAATCTTGCTCAGCTTGTC-3′ |
| ZEB1 primer | Forward:5′-ACCCTTGAAAGTGATCCAGC-3′ |
|  | Reverse:5′-CATTCCATTTTCTGTCTTCCGC-3′ |
| ZEB2 primer | Forward:5′-TCCAGAAAAGCAGTTCCCTTC-3′ |
|  | Reverse:5′-CACACTGATAGGGCTTCTCG-3′ |
| siNC | Sense: 5′-UUCUCCGAACGUGUCACGUTT-3′ |
|  | Antisense: 5′-ACGUGACACGUUCGGAGAATT-3′ |
| siPTPRM-1 | Sense: 5′-GGAUACAGCUCAACGCCAATT-3′ |
|  | Antisense: 5′-UUGGCGUUGAGCUGUAUCCTT-3′ |
| siPTPRM-2 | Sense: 5′-CCAUACAAGGAAGUACCUUTT-3′ |
|  | Antisense: 5′-AAGGUACUUCCUUGUAUGGTT -3′ |
| siVEGF-C | Sense: 5′-GCCGAUGCAUGUCUAAACUTT-3′ |
|  | Antisense: 5′-AGUUUAGACAUGCAUCGGCTT-3′ |

**Table S2 Antibodies used in the study**

| **Antibody** | **Catalog#** | **Working concentration** | **Manufacturer** |
| --- | --- | --- | --- |
| PTPRM (WB) | 436502 | 1ug/ml | R&D |
| PTPRM (IHC) | ab231607 | 20 ug/ml | Abcam |
| GAPDH | AP0063 | 1:5000 | Bioworld |
| E-cadherin | 20874-1-AP | 1:5000 | Proteintech |
| N-cadherin | 22018-1-AP | 1:2500 | Proteintech |
| Vimentin | 5741 | 1:1000 | CST |
| VEGF-C | sc-374628 | 1:1000 | Santacruz |
| Ki-67 | 23709-1-AP | 1:6000 | Proteintech |
| AKT | 4691 | 1:1000 | CST |
| pAKT | 4060 | 1:2000 | CST |
| Src | 2123 | 1:1000 | CST |
| Non-pY529Src | 2107 | 1:1000 | CST |
| pY418Src | 6943 | 1:1000 | CST |
| Bax | 5023 | 1:1000 | CST |
| Bcl-2 | 4223 | 1:1000 | CST |
| Cleaved caspase-9 | 9502 | 1:1000 | CST |
| Cleaved caspase-3 | 9661 | 1:1000 | CST |
| LYVE-1 | ab14917 | 1:50 | Abcam |
| Snail | 3879 | 1:1000 | CST |

**Table S3 Popliteal lymph node metastasis in vivo**

| Group | No. Total LNs | No. Metastasis LNs | Metastatic ratio (%) | *P* value |
| --- | --- | --- | --- | --- |
| SiHa-shNC | 10 | 5 | 50 | *P* = 0.141 |
| SiHa-shPTPRM | 10 | 1 | 10 |  |
| HeLa-shNC | 10 | 4 | 40 | *P* = 0.087 |
| HeLa-shPTPRM | 10 | 0 | 0 |  |
